# Identification of a Prognostic Signature for Ovarian Cancer Based on Ubiquitin-Related Genes Suggesting a Potential Role for FBXO9

**DOI:** 10.3390/biom13121724

**Published:** 2023-11-30

**Authors:** Xiaomei Luo, Yingjie Wang, Hao Zhang, Guangquan Chen, Jindan Sheng, Xiu Tian, Renhao Xue, Yu Wang

**Affiliations:** 1 Department of Gynecology, Shanghai First Maternity and Infant Hospital, School of Medicine, Tongji University, Shanghai 200092, Chinachenguangquan@tongji.edu.cn (G.C.);; 2 Shanghai Key Laboratory of Maternal Fetal Medicine, Shanghai Institute of Maternal-Fetal Medicine and Gynecologic Oncology, Clinical and Translational Research Center, Shanghai First Maternity and Infant Hospital, School of Medicine, Tongji University, Shanghai 200092, China; 3 School of Life Sciences and Technology, Tongji University, Shanghai 200092, China

**Keywords:** ovarian cancer, ubiquitination, prognostic signature, *FBXO9*, *UBD*, DNA damage repair

## Abstract

Background: Ovarian cancer (OV) is associated with high mortality and poses challenges in diagnosis and prognosis prediction. Ubiquitin-related genes (UbRGs) are involved in the initiation and progression of cancers, but have still not been utilized for diagnosis and prognosis of OV. Methods: K48-linked ubiquitination in ovarian tissues from our OV and control cohort was assessed using immunohistochemistry. UbRGs, including ubiquitin and ubiquitin-like regulators, were screened based on the TCGA-OV and GTEx database. Univariate Cox regression analysis identified survival-associated UbRGs. A risk model was established using the LASSO regression and multivariate Cox regression analysis. The relationship between UbRGs and immune cell infiltration, tumor mutational burden, drug sensitivity, and immune checkpoint was determined using the CIBERSORT, ESTIMATE, and Maftools algorithms, based on the Genomics of Drug Sensitivity in Cancer and TCGA-OV databases. GEPIA2.0 was used to analyze the correlation between *FBXO9*/*UBD* and DNA damage repair-related genes. Finally, *FBXO9* and *UBD* were accessed in tissues or cells using immunohistochemistry, qPCR, and Western blot. Results: We confirmed the crucial role for ubiquitination in OV as a significant decrease of K48-linked ubiquitination was observed in primary OV lesions. We identified a prognostic signature utilizing two specific UbRGs, *FBXO9* and *UBD*. The risk score obtained from this signature accurately predicted the overall survival of TCGA-OV training dataset and GSE32062 validation dataset. Furthermore, this risk score also showed association with immunocyte infiltration and drug sensitivity, revealing potential mechanisms for ubiquitination mediated OV risk. In addition, *FBXO9*, but not *UBD*, was found to be downregulated in OV and positively correlated with DNA damage repair pathways, suggesting *FBXO9* as a potential cancer suppressor, likely via facilitating DNA damage repair. Conclusions: We identified and validated a signature of UbRGs that accurately predicts the prognosis, offers valuable guidance for optimizing chemotherapy and targeted therapies, and suggests a potential role for *FBXO9* in OV.

## 1. Introduction 

Ovarian cancer (OV) is a leading cause of gynecological malignancy mortality [1]. There are 19,710 newly diagnosed cases of OV each year in the United States, with 13,270 death cases [2]. Moreover, the vast majority of OV patients are diagnosed at advanced stages due to a lack of early symptoms [3]. Meanwhile, for patients with middle-advanced stage OV, five-year overall survival is no more than 30–50% [4]. Such a high mortality is largely attributed to the lack of diagnostic biomarkers. Additionally, approximately 70% of patients have a recurrence within 3 years [1], so the prediction of prognosis is currently a major challenge for OV. Therefore, developing a novel and promising biomarker for diagnosis and prognosis is a priority for current OV study.

Ubiquitination is a ubiquitous post-translational modification of proteins in eukaryotic cells. Covalent linkage of monoubiquitins or polyubiquitin chains to one or more specific amino acid sites modulates a variety of functions of the substrate protein, depending on diverse topology of the chain linkage [5]. The most prominent role of ubiquitination is to drive proteasomal degradation of the substrate protein, usually through a specific type of polyubiquitin chain with K48-linkages [6]. Ubiquitination-mediated protein stability, localization, and functions have been reported to affect multifarious vital life processes such as autophagy, DNA damage repair, cell cycle, signal transduction, gene expression, inflammation, immunity, etc. [7,8] Recent studies have also revealed the crucial roles of ubiquitination in cancer during tumorigenesis, tumor development, and metastasis [9,10]. For example, ubiquitin-related pathways were found to augment anti-tumor immunity by enhancing T-cell activation [11]. As a result, novel anti-cancer therapies targeting ubiquitin-related genes (UbRGs) are emerging, and direct targeting of ubiquitin potentially boosts the effect of T-cell immunotherapy [12,13].

Previous studies have demonstrated the pivotal involvement of UbRGs in OV. A series of deubiquitinating enzymes (DUBs) that remove ubiquitin from substrate proteins were found to be overexpressed in OV and demonstrated oncogenic properties [14]. Several ubiquitin and ubiquitin-like conjugases and ligases showed altered expression in low malignant potential and high-grade serous ovarian cancer [15]. Therefore, UbRGs are likely to play a critical role in OV. However, functions of UbRGs have not been systematically studied. The value of UbRGs in diagnosis and treatment of OV remains undetermined. This study aims to investigate the potential association between ubiquitin-related genes and mechanism underlying in OV.

In this study, we found that K48-linked ubiquitination, which leads to protein degradation via ubiquitin-proteasome pathway, was significantly downregulated in OV based on a cohort of 88 patients, evidencing the involvement of ubiquitination in OV. Hence, we further developed an expression-based prognostic signature of UbRGs based on analysis of gene expression profiles of OV patients from The Cancer Genome Atlas (TCGA) dataset as well as normal controls from the Genotype-Tissue Expression Project (GTEx) dataset. This prognostic signature also exhibited good performance in predicting immune cell infiltration in the tumor microenvironment (TME) and drug sensitivity. Furthermore, *FBXO9*, one UbRG within this signature, is downregulated in OV patients and cell lines, associates with DNA damage repair activity, and impacts the survival and proliferation of cancer cell lines, suggesting a potential role of *FBXO9* in tumorgenesis of OV. In all, our findings provided a novel two-gene prognostic signature for OV and further revealed the role of FBXO9-related ubiquitination in OV.

## 2. Materials and Methods 

### 2.1. Patients and Specimens

All patients with OV were admitted to Shanghai First Maternity and Infant Hospital from 1 May 2020 to 1 May 2023. We obtained primary ovarian cancer samples from 88 patients with OV. A total of 28 ovarian tissue samples were obtained from patients with benign gynecologic diseases. These samples were used as normal ovarian controls as they exhibited typical nontumorous morphology by Hematoxylin and Eosin (HE) staining (Figure S1). Next, all tissue samples were made into tissue microarrays. Medical records were used to gather clinicopathological data. All patients provided written informed consent.

### 2.2. Immunohistochemistry (IHC)

The IHC scoring criteria were adopted from established protocols. The following reagents were used: Triton X-100 (Beyotime, Shanghai, China), Immunol staining blocking buffer (Beyotime, China), anti-fluorescence quenching (Solarbio, Beijing, China), GTVisionTM Anti-mouse/Anti-rabbit Immunohistochemical Analysis Kit (KeyGEN BioTECH, Nanjing, China, GK500705), Anti-Ubiquitin linkage-specific K48 (Abcam, Cambridge, UK, ab140601), and FBXO9 Rabbit Polyclonal Antibody (ORIGENE, Rockville, MD, USA, TA368310S). Only stained tissues with a clear ovary morphology were used for analysis. Staining intensity was assessed using the following scale: 0 (no staining), 1 (weak staining), 2 (moderate staining), and 3 (strong staining). The proportion of positive tumor cells was evaluated using the following scale: 1 (<10%), 2 (10–35%), 3 (35–70%), and 4 (>70%). The immunoreactivity scores (IRS) were calculated by multiplying the staining intensity score with the percentage score of positive tumor cells. Scores ranging from 0 to 5 were considered negative, while scores higher than 5 were classified as positive expressions [16].

### 2.3. Data Extraction and Patient Information Precondition

Figure S2 presents the flowchart illustrating the sequential process of our research study. Transcriptome profiles and clinical characteristics of TCGA-OV patients (*n* = 375) were obtained from UCSC Xena (https://xenabrowser.net/, accessed on 11 September 2023) and converted from Fragments Per Kilobase of transcript per Million mapped reads to log2 (TPM (Transcripts Per Million) + 1). Patients lacking clinical pathological data or overall survival (OS) were excluded from the study. The transcriptome profiles of normal ovarian tissue (*n* = 88) were obtained from the GTEx database (https://www.gtexportal.org/, accessed on 11 September 2023). The “limma” package was used for removing batch effects. For external validation, we downloaded GSE32062 (*n* = 260, Platform = GPL6480) matrix files, along with basic clinical and survival information, from the GEO database (https://www.ncbi.nlm.nih.gov/geo/, accessed on 11 September 2023) for independent datasets.

### 2.4. Screening and Annotation of Ubiquitin-Related Genes

We retrieved a total of 1366 UbRGs from the iUUCD 2.0 database [17]. The ubRGs include regulators of ubiquitin and ubiquitin-like conjugations. mRNA expression profiles of 780 ubiquitin-related genes were obtained from the TCGA database. We identified 287 UbRGs differentially expressed between normal and tumor samples using the “limma” R package, based on|log2 Folding change (FC)| > 1 and adjust *p* < 0.05. A volcano plot was constructed based on fold change and adjusted P-value, and a heatmap was generated to visualize differential gene expression.

We uploaded all differentially expressed-ubiquitin-related genes (DE-UbRGs) to the Search Tool for the Retrieval of Interacting Genes (https://string-db.org/, accessed on 11 September 2023) and visualized them in Cytoscape with a minimum interaction score of 0.4. Cytoscape software (version 3.10.1) was used to construct the protein–protein interaction (PPI) network and analyze the interactions of the DE-UbRGs. The CytoHubba plugin selects the top 30 DE-UBRGs as hub genes based on the highest degree value sorting, aiming to reveal protein interactions. Functional analysis of DE-UbRGs was implemented using Gene Ontology (GO) and Kyoto Encyclopedia of Genes and Genomes (KEGG) through the R package “ClusterProfiler” to determine their potential functions. The functional enrichment results were filtered using a significance threshold of *p* < 0.05.

### 2.5. Construction and Validation of a Prognostic Signature Based on Ubiquitin-Related Genes

Univariate Cox regression analysis was conducted to identify UbRGPs associated with OS within the TCGA training cohort. Following this, we employed the least absolute shrinkage and selection operator (LASSO) Cox regression algorithm and a stepwise multivariate Cox proportional hazard regression analysis to formulate a prognostic signature based on UbRGs within the training cohort. Patients were stratified into two groups based on their median risk scores using a multivariate Cox model. Receiver operating characteristic (ROC) curve, survival curve, survival status, and risk score distribution were analyzed.

### 2.6. Construction of Calibration Curves and Nomograms

The “RMS” package was utilized to construct a nomogram for assessing the survival probability of individuals, considering their risk scores and other clinicopathological information. The survival probability of each patient is determined based on the cumulative score of all factors [18]. Calibration curves were employed to evaluate the concordance between predicted and observed survival outcomes. ROC analysis was employed to assess the predictive performance of the nomogram for survival.

### 2.7. Analysis of Tumor Immune Microenvironments and Somatic Mutations

The “ESTIMATE” package utilizes gene expression data to assess the cellular composition within the sample. The “ESTIMATE” package was utilized to calculate estimate, stromal, and immune scores for all OV samples [19]. To comprehensively evaluate the immune infiltration landscape, we employed CIBERSORT algorithms to assess the presence of 22 representative immune cell types within the TME [20]. To identify effective immunotherapy for OV patients, we assessed immune checkpoint gene expression (including *CTLA4*, *CD274*, *HAVCR2*, *LAG3*, *PDCD1LG2*, *PDCD1*, *TIGIT*, and *SIGLEC15*). The total number of somatic coding errors, base substitutions, and indel mutations per megabase is referred to as the tumor mutational burden (TMB). A waterfall plot generated from the TCGA database, utilizing the “Maftools” R package, illustrates the 20 most frequently occurring tumor mutation genes. Each column corresponds to an individual patient.

### 2.8. Predicting the Susceptibility to Chemotherapy Agents

Each sample was assessed for drug sensitivity using “oncoPredict” package to determine the IC50 values for commonly used targeted therapy agents and chemotherapy agents. IC50 values were obtained from the Genomics of Drug Sensitivity in Cancer database (GDSC, https://www.cancerrxgene.org/, accessed on 11 September 2023).

### 2.9. Analysis of the FBXO9 and UBD Interaction Network and Functional Enrichment

To investigate the association between *FBXO9*, *UBD*, and DNA damage repair (DDR), including base excision repair (BER), Homologous Recombination repair (HRR), and mismatch repair (MMR) [21], we identified a set of genes related to DNA damage repair and analyzed their correlation with *FBXO9* and *UBD* using GEPIA2.0. Afterward, we plotted the correlation heatmap between DNA damage repair related genes and *FBXO9* and *UBD. FBXO9* expression was examined using GEPIA2.0, and through its co-expression analysis function [22], we identified the top 100 genes that are co-expressed with *FBXO9* in OV. Functional analysis of the genes was performed using the ‘clusterProfiler’ R package, with a significance threshold of *p* < 0.05. To gain further insights into the function of *FBXO9,* we stratified all cancer samples into low and high *FBXO9* groups based on the median expression of *FBXO9*. Additionally, we performed Gene Set Enrichment Analysis (GSEA) to further investigate the functional enrichment of *FBXO9* [23].

### 2.10. Cell Lines

Human ovarian cancer cell lines, including A2780, SKOV-3, HEY, HOC7, IGROV1, OVCAR-8, TOV-21G, and OVCAR-3, as well as one normal ovarian epithelial cell line IOSE80, were acquired from the American Type Culture Collection (ATCC, Manassas, VA). The cells were cultured in Dulbecco’s Modified Eagle’s Medium supplemented with 10% fetal bovine serum and 1% penicillin/streptomycin. Regular testing was performed to authenticate the cells and confirm their mycoplasma-free status. The Clustered Regularly Interspaced Short Palindromic Repeats (CRISPR) data for *FBXO9*, *UBD*, *BRCA1*, and *BRCA2* in 317 cancer cell lines including 29 ovarian cancer cell lines, and the protein abundance data for Ki-67 in 8 ovarian cancer cell lines were downloaded from the Cancer Cell Line Encyclopedia (CCLE, https://depmap.org/portal/download/all/?releasename=CCLE+2019, accessed on 11 September 2023) database. The CERES project score and the relative protein level were used for analysis. 

### 2.11. RNA Extraction and Gene Expression Analysis

Total RNA was isolated from ovarian cells and tissues using Trizol Reagent (Invitrogen™, Carlsbad, CA, USA) following the manufacturer’s protocol. The primer sequences are listed in Table S1. The reagents used included the PrimeScriptTM RT Reagent Kit (TaKaRa, Shiga, Japan, RR820A) and TB Green Premix Ex TaqTMII (TaKaRa, Shiga, Japan, RR820A).

### 2.12. Western Blot (WB) Assay

The experimental technique of WB was carried out in accordance with established protocols [24]. Anti-FBXO9 (ORIGENE, Rockville, MD, USA, TA368310S) was used as the primary antibody.

### 2.13. Statistical Analysis

Statistical analyses were conducted using R software (version 4.2.3) and GraphPad Prism software (version 9), following the methods described above. Statistical significance was defined as *p* < 0.05 for all analyses.

## 3. Results

### 3.1. K48-Linked Ubiquitination was Suppressed in OV

**Figure 1:**
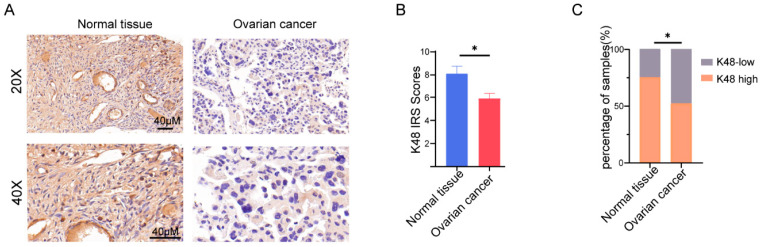
Downregulation of K48-linked ubiquitination in OV. (**A**) Representative images showing IHC staining of K48-linked polyubiquitin chains for normal tissue of ovary or primary lesion of ovarian cancer. (**B**) Quantification of K48-linked ubiquitination using the IRS score. IRS scores from normal tissue of ovary and primary lesion of ovarian cancer were plotted as mean ± SEM. (**C**) The proportions of low (orange, IRS ≤ 5) versus high (purple, IRS > 5) K48-linked ubiquitination were plotted. * *p* < 0.05, unpaired *t*-test or kappa square test.

Although crucial functions of protein ubiquitination have been identified for decades, the involvement of ubiquitin-proteasome pathway in OV is still unclear. Hence, we studied the ubiquitination-mediated protein degradation in a cohort of 88 OV patients and 28 women with normal ovaries as the control. As K48-linked ubiquitination is a canonical signal of ubiquitin-proteasome dependent protein degradation [25], ovarian carcinoma or control tissues were IHC stained using an antibody specifically targeting K48-linked polyubiquitin chains to estimate the activity of ubiquitin-proteasome pathway. Strikingly, the abundance of K48-linked polyubiquitin chains showed a robust decrease in primary ovarian lesions as compared to normal ovary tissues (Figure 1A). The level of IHC staining was quantified using IRS. The IRS obtained from the OV primary lesion (5.944 ± 0.4162, *n* = 83, 5 samples were removed due to incorrect morphology) was significantly lower than that from normal tissue (8.06 ± 0.6828, *n* = 28) (Figure 1B). A total of 75% of normal ovary tissues (21/28) exhibited a high level of K48-linked polyubiquitin chains whereas only 51.8% of primary OV lesions (43/83) demonstrated high activity of K48-linked ubiquitination (IRS > 5) (Figure 1C). Western blot assay of 4 control and 7 OV tissue samples also yielded consistent results (Figure S3A). No significant difference between high and low K48-linked polyubiquitination OV patients was observed for clinicopathological characteristics, including age, FIGO stage, pathology stage, histology type, tumor diameter, and serum CA-125 levels (*p* ≥ 0.05; Table S2). These findings indicated that suppression of the ubiquitin-proteasome pathway and subsequent misregulation of protein degradation might contribute to the tumorigenesis and development of OV, suggesting ubiquitination as a potential marker for the diagnosis and prognosis of OV.

### 3.2. Identification of 287 DE-UbRGs Involved in OV

**Figure 2:**
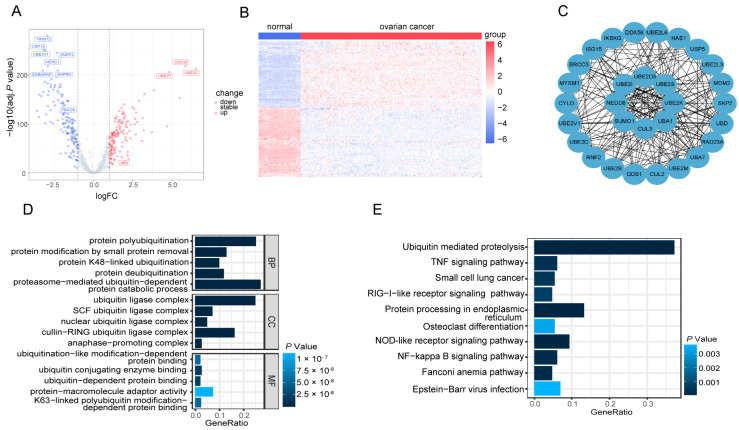
Identification of DE-UbRGs in OV. (**A**) The volcano plot of 287 DE-UbRGs was constructed concerning the adjusted P values and fold change (FC) values. The red dots indicate up-regulated genes, while the blue dots indicate downregulated genes (|log_2_(FC)|>1 and adjusted *p* < 0.05). (**B**) The heatmap of DE-UbRGs in normal (GTEx dataset, blue) and OV tumor (TCGA-OV dataset, red). (**C**) The PPI network diagram of the top 30 DE-UbRGs. (**D**,**E**) GO (**D**) and KEGG (**E**) pathway enrichment analysis of DE-UbRGs. BP: Biological Process; CC: Cellular Component; MF: Molecular Function).

To utilize ubiquitination as an OV biomarker, we conducted a series of studies to isolate UbRGs expression signature from 780 UbRGs (Figure S2). To be noted, this collection of UbRGs consists of all regulators that modulate ubiquitin and ubiquitin-like conjugations [17] and is not limited to K48-linked ubiquitination. Firstly, gene expression profiles of the TCGA-OV cohort (*n* = 375) along with the GTEx cohort (*n* = 88) as control subjects were screened for all 780 UbRGs. In total, 287 DE-UbRGs in OV versus normal samples were identified, as revealed by the volcano plot (Figure 2A) and highlighted by the heatmap (Figure 2B). We also established a PPI network among the top 30 DE-UbRGs with the highest degree of identification (see Section 2), using the Search Tool for Retrieval of Interacting Genes database (https://string-db.org) with the CytoHubba plugin. This analysis aimed to unveil protein interactions that could offer valuable insights for investigating potential underlying mechanisms (Figure 2C). GO and KEGG pathway enrichment analysis were conducted for the DE-UbRGs, and the top 15 most significant GO as well as top 10 most significant KEGG pathways were shown in Figure 2D,E. As expected, ubiquitination related pathways, including ubiquitin-mediated proteolysis, and protein K48-linked ubiquitination, exhibited significant enrichment. Moreover, cancer related pathways, such as TNF signaling and NF-kappa B signaling, were also enriched. Interestingly, we also found the enrichment of Fanconi anemia pathway, suggesting the involvement of DNA damage repair for interstrand crosslink [26].

### 3.3. Construction and Estimation of a Prognostic Signature of UbRGs for OV

**Figure 3:**
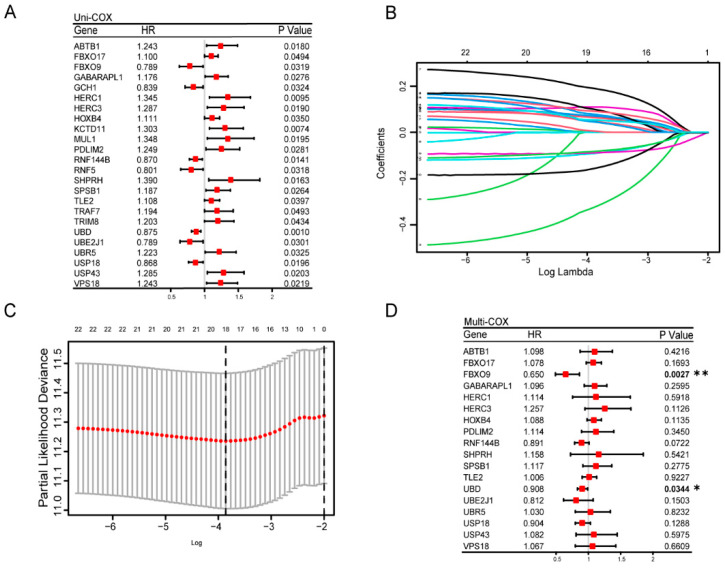
Construction of a prognostic risk model for OV based on ubiquitination. (**A**) Univariate Cox proportion hazards regression. (**B**) The λ selection diagram for the LASSO tuning parameter selection, with 10-fold cross-validation. Each colored line represents the inclusion of 24 different genes and show the Log Lambda value corresponding to the minimum cross-validation error point. (**C**) The LASSO-Cox analysis for 24 optimal prognostic UbRGs. (**D**) Multivariate Cox regression analysis showed two significant (*p* < 0.05) genes: *FBXO9* and *UBD*. * *p* < 0.05, ** *p* < 0.01.

Using the implementation of univariate Cox regression analysis for 287 DE-UbRGs, we further yielded 24 genes that showed association with OS (Figure 3A). A LASSO regression model was constructed, utilizing features obtained from the univariate Cox analysis, which contributed to creating a more simplified model. Through LASSO regression analysis, the optimal lambda value (0.02105545) was selected, simplifying 24 features into 18 potential prognostic factors (Figure 3B,C). To further improve explicability, we performed a multivariate Cox regression analysis and finally identified two genes, namely *FBXO9* and *UBD*, as a prognostic signature (Figure 3D). *FBXO9* encodes an E3 ubiquitin ligase [27] while *UBD* encodes FAT10, which is a Ubiquitin-like protein modifier [28]. Ultimately, the risk score was calculated in the multivariate Cox proportional hazard model, as shown by the following equation:

risk score=(−0.2051)×FBXO9+(−0.1272)×UBD

**Figure 4:**
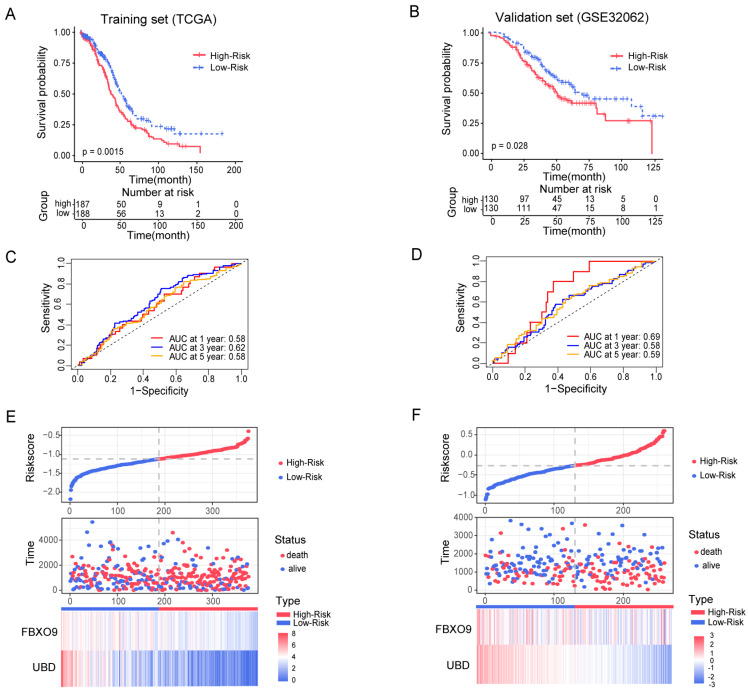
Stability and predictive power of the prognostic risk model. (**A**,**B**) The OS curves for high-risk versus low-risk OV patients of the TCGA-OV training set (**A**) and GSE32062 validation set (**B**) were plotted using K-M analysis, respectively. The *p* values of K-M test are shown in number. (**C**,**D**) The ROC analysis for the OS prediction value of the ubiquitin-related signature for the TCGA-OV training set (**C**) and GSE32062 validation set (**D**) were shown, respectively. The AUC values are shown in number. (**E**,**F**) The scatter plots of risk scores (top) and the survival time (months) with status (middle), and the heatmaps demonstrating expression profiles of gene *FBXO9* and *UBD* (bottom) were shown for each OV patient from the TCGA-OV training set (**E**) and GSE32062 validation set (**F**).

The risk score for ovarian cancer patients was calculated for both the training cohort (TCGA-OV set; *n* = 375) and the validation set (GSE32062 set; *n* = 260), according to the formula provided above. Using median as cut-off value, we categorized OV patients into two risk groups: low-risk and high-risk. Kaplan–Meier (K-M) analysis demonstrated that low-risk patients had significantly improved OS in both training cohort (*p* = 0.0015) (Figure 4A) and validation cohort (*p* = 0.028) (Figure 4B). The prognostic value of this ubiquitination related signature was further approved via a time-dependent ROC analysis for 1-year, 3-year, and 5-year OS in both the training (Figure 4C) and validation (Figure 4D) cohort. In addition, Figure 4E,F visualized consistency of risk score, survival time, and status, along with the expression profile of the two ubiquitin-related signature genes, *FBXO9* and *UBD.*

### 3.4. Construction and Validation of a Ubiquitin-Related Prognostic Nomogram

**Figure 5:**
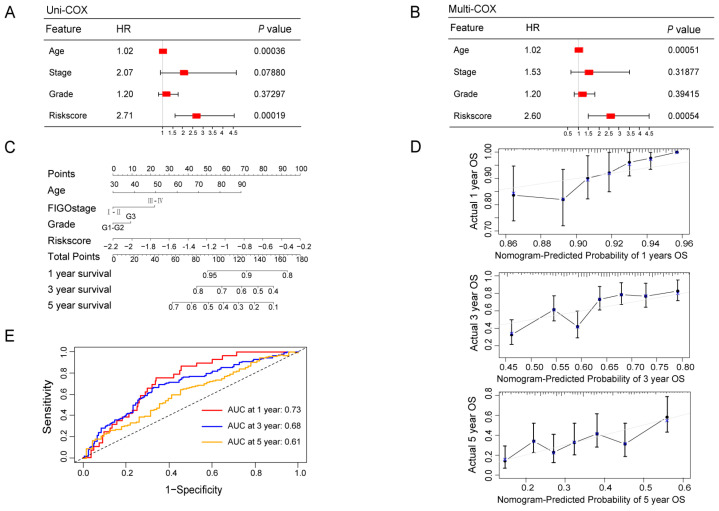
Establishment and evaluation of a nomogram for OV patients in the TCGA cohort. The forest plots presented the univariate (**A**) and multivariate Cox Hazard Regression (**B**) analysis for OV patient survival, based on the ubiquitin-related 2-gene signature and clinical features (including age, FIGO stage, and grade). (**C**) The prognostic nomogram model was conducted to predict the 1-year, 3-year, and 5-year OS of OV patients, based on the ubiquitin-related signature and clinical indicators (namely age, FIGO stage, Grade). (**D**) Calibration curves were plotted to assess the performance of the prognostic nomogram model in predicting 1-year (top), 3-year (middle), and 5-year (bottom) OS. The gray line represents the ideal nomogram, while the blue lines depict the observed nomogram. The closer the solid gray line is to the blue line, the more accurately the model predicts survival. (**E**) ROC curves of the nomogram.

To identify prognostic indicators for OV patients, we performed both univariable and multivariable Cox Regression analyses, which revealed that age (*p* = 0.00036) and risk score (*p* = 0.00019) were independent prognostic factors (Figure 5A,B). We determined the OS of patients at 1, 3, and 5 years by creating a nomogram based on the ubiquitin-related signature and clinical indicators (namely age, FIGO stage, and grade) (Figure 5C). The accuracy and sensitivity of the prediction were confirmed using a calibration chart of the nomogram. The calibration curve demonstrated a good match between the actual and predicted survival rates at 1-, 3-, and 5-year intervals (Figure 5D). We aim to investigate the predictive ability of the above-mentioned model. The ROC curve revealed that the area under the curve (AUC) of the nomogram was 0.73 at 1 year, 0.68 at 3 years, and 0.61 at 5 years (Figure 5E). The results indicated that the nomogram exhibited moderate accuracy in predicting OS.

### 3.5. OV patients Subgrouped Using the Ubiquitin-Related Signature Demonstrate Distinct TME Characteristics

**Figure 6:**
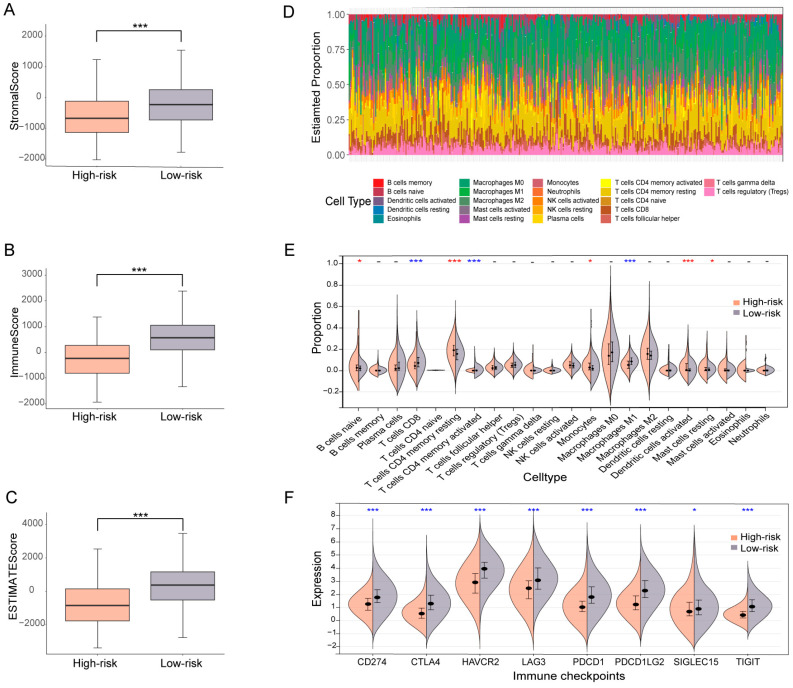
Characterization of cellular composition and immune cell infiltration profile of TME for high-risk versus low-risk OV patients. (**A**–**C**) The stromal score (**A**), immune score (**B**), and ESTIMATE score (**C**) of the high-risk versus low-risk group were plotted as boxplots. (**D**) The heatmap represented the composition of the 22 typical immune cell infiltration in the TME of OV samples in both low-risk and high-risk groups, based on the CIBERSORT algorithm. (**E**) The violin plots with mean ± SEM showed the composition of the 22 typical immune cell infiltration in the 2 risk groups, which were stratified by the ubiquitin-related signature. (**F**) The violin plots with mean ± SEM showed the expression difference of eight typical immune checkpoints (including *CD274*, *CTLA4*, *HAVCR2*, *LAG3*, *PDCD1*, *PDCD1LG2*, *SIGLEC15*, and *TIGIT*) between the two risk groups stratified by the UbRGs. n.s. *p* > 0.05, * *p* < 0.05, *** *p* < 0.001, unpaired *t*-test (red represents a higher proportion of high-risk groups and blue represents a lower proportion of high-risk groups).

As the above findings have highlighted the critical role of ubiquitin-related genes in OV, the mechanism that underlies the ubiquitination mediated OV risk remains to be explored. Firstly, analysis of gene mutations revealed no significant difference in the TMB and large overlap in the top 20 mutated genes (9 out of 20) for high-risk versus low-risk groups (Figure S4A,B), suggesting that intrinsic genetical alterations might not be the major mechanism for ubiquitination related cancer progress. Hence, we propose that the mediation of OV risk by ubiquitin-related genes is largely through modulation of TME. To test this hypothesis, we scored the cell composition of TME for either low-risk or high-risk samples using the ESTIMATE analytical package. Remarkably, the low-risk group exhibited significantly higher scores in stromal, immune, and ESTIMATE categories (Figure 6A–C), indicating different cellular constituents of TME for patients with distinct ubiquitin-related signatures. Given a significantly lower immune score in the high-risk group, we further evaluated the immune infiltration landscape to examine the association between ubiquitination and the tumor immune microenvironment. The infiltration status of 22 typical types of immune cells in TME was profiled for both low-risk and high-risk OV samples, utilizing the CIBERSORT algorithm (Figure 6D). Interestingly, the high-risk group demonstrated significantly decreased infiltration for three cell types (T cells CD8, T cells CD4 memory activated, and macrophages M1) that are key anti-cancer immune cells, increased for another 5 cell types (B cells naïve, T cells CD4 memory resting, monocytes, dendritic cells activated, and mast cells resting), and unchanged for the rest 14 cell types (Figure 6E), suggesting a nonidentical tumor immune microenvironment. Furthermore, altered infiltration of immune cells conceivably results from different expressions of immune checkpoint genes. According to the RNA-sequencing expression profiles of the TCGA-OV cohort, we thereby conducted the association analysis between immune checkpoint expression and the ubiquitin-related signature, indicating that *CD274*, *CTLA4*, *HAVCR2*, *LAG3*, *PDCD1*, *PDCD1LG2*, *SIGLEC15*, and *TIGIT* were significantly up-regulated in the low-risk group (*p* < 0.05). Thus, OV patients in the low-risk group were more likely to benefit from immunotherapies targeting these eight checkpoints (Figure 6F).

### 3.6. High Risk Based on the Prognostic Signature Corresponds with High Drug Resistance

**Figure 7:**
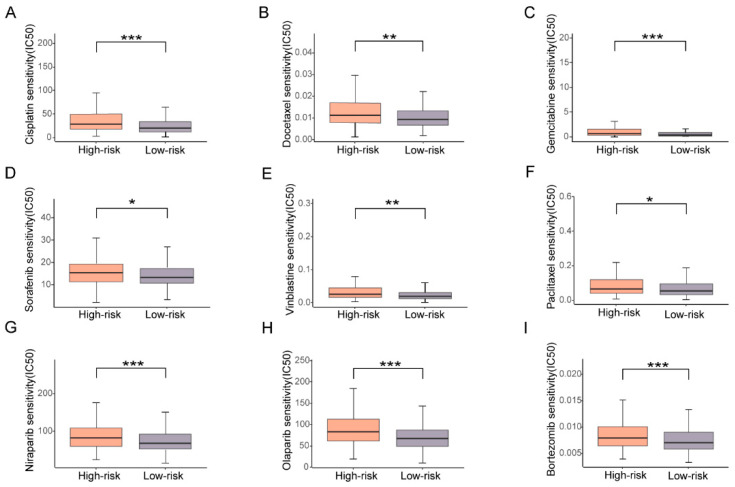
Drug sensitivity (IC50) of high-risk versus low-risk OV patients. (**A**–**I**) The boxplots represented the estimated IC50 values of OV patients towards 6 typical chemotherapies, including Cisplatin (**A**), Docetaxel (**B**), Gemcitabine (**C**), Sorafenib (**D**), Vinblastine (**E**), Paclitaxel (**F**), PARP inhibitors, including Niraparib (**G**), and Olaparib (**H**), and proteasomal inhibitor Bortezomib (**I**). The targeted therapy sensitivity analyses were carried out through the GDSC. * *p* < 0.05, ** *p* < 0.01, *** *p* < 0.001, unpaired *t*-test.

Different OS could result from distinct drug sensitivity, and TME also affects sensitivity to anti-cancer medicine. Hence, we investigated the relationship between ubiquitin-related prognostic signature and sensitivity to drugs of chemo- or targeted therapy. The present study determined the half-maximal inhibitory concentration (IC50) values of six common chemotherapies (cisplatin, docetaxel, gemcitabine, paclitaxel, sorafenib, vinblastine), two PARP inhibitors (niraparib, olaparib), and one proteasome inhibitor (bortezomib [29]), based on data from the GDSC database. Patients in the low-risk group exhibited significantly higher sensitivity (lower IC50) to all drugs used (Figure 7A–H). Interestingly, bortezomib, one of proteasome inhibitors employed in clinical practice, also presented a favorable response in the low-risk group (Figure 7I). These results indicated that the risk according to the ubiquitin-related prognostic signature is associated with resistance to commonly used drugs for OV.

### 3.7. FBXO9 Is Involved in DNA Damage Repair

**Figure 8:**
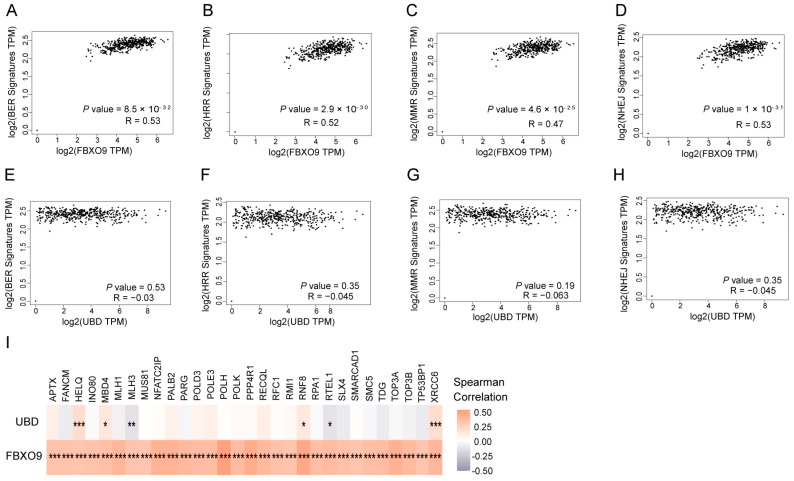
Relationship of *FBXO9* and *UBD* with DNA damage repair pathways in OV. (**A**–**H**) The correlation scatter plots in ovarian cancer present the correlations between *FBXO9* (**A**–**D**) or *UBD* (**E**–**H**) mRNA expression and 4 individual DNA damage repair signatures, including the BER signature of 47 genes (**A**,**E**), the HRR signature of 88 genes (**B**,**F**), the MMR signature of 24 genes (**C**,**G**), and the NHEJ signature of 23 genes (**D**,**H**). (**I**) The heatmap displays the associations between *FBXO9/UBD* and DDR genes in OV (R > 0.3). * *p* < 0.05, ** *p* < 0.01, *** *p* < 0.001, Spearman’s rank correlation coefficient or unpaired *t*-test.

UbRGs are associated with tumor immune microenvironment and drug sensitivity, which might affect the progression of OV [30]. However, it is still questionable how UbRGs suppress the occurrence of OV. For OV and many other types of cancer, dysfunction of DDR and subsequential enhancement of genomic instability, i.e., *BRCA1/2* gene mutation induced DDR deficiency, is a key etiology of tumorigenesis [31]. Interestingly, our KEGG analysis demonstrated enrichment of the Fanconi anemia DNA interstrand crosslink repair pathway (Figure 2E). Taken together, we speculate that ubiquitination might control OV occurrence via modulation of DNA repair pathways. To test this hypothesis, functional enrichment analyses were performed to determine the correlations of *FBXO9* and *UBD*, respectively, with a series of DNA repair gene signatures, including BER, HRR, MMR, and NHEJ signatures. We found that *FBXO9* was positively correlated with all four groups of DNA damage repair related genes (Figure 8A–D), while *UBD* was not associated with any of the four DNA damage pathways mentioned above (Figure 8E–H). We further observed a significant positive correlation between *FBXO9* and DDR genes, with Spearman’s correlation coefficients exceeding 0.3. By contrast, there is only a poor correlation between *UBD* and DDR genes (Figure 8G). This finding established *FBXO9*, but not *UBD*, as a positive mediator of DNA repair, which potentially impairs genomic instability and cancer occurrence. In addition, besides DNA repair, *FBXO9* might contribute to some other tumorigenesis related pathways. The top 100 *FBXO9* co-expressed genes in OV were analyzed using GEPIA2.0. The functional enrichment of KEGG terms exhibited TNF signaling pathway and cell cycle (Figure S5A). Moreover, the GSEA results suggested the close association between choline metabolism and ECM-receptor interaction with *FBXO9* expression (Figure S5B).

### 3.8. Altered Expression Level of FBXO9 in OV

**Figure 9:**
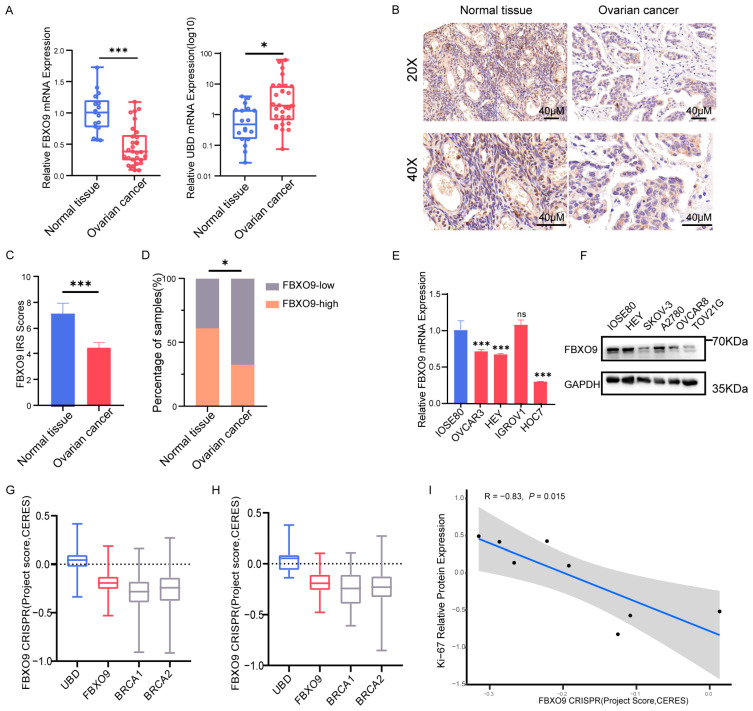
Alteration of *FBXO9* and *UBD* expression in OV. (**A**) The relative *FBXO9* and *UBD* mRNA expression levels in ovarian tissues from OV or control patients were determined by qRT-PCR and plotted as scatter plots with boxplots. Each point represents the mean of three independent biological repeats. (**B**) Representative images showing IHC staining of FBXO9 for normal tissue of ovary or primary lesion of ovarian cancer. (**C**) Quantification of FBXO9 using IRS score. IRS scores from normal tissue of ovary and primary lesion of OV were plotted as mean ± SEM. (**D**) The proportions of low (orange, IRS ≤ 5) versus high (purple, IRS > 5) FBXO9 were plotted. ^n.s.^ *p* > 0.05, * *p* < 0.05, *** *p* < 0.001, unpaired *t*-test or kappa square test. (**E**) The relative *FBXO9* mRNA expression levels in different cell lines were determined by qRT-PCR and plotted as mean ± SEM. Data were from three independent biological repeats. (**F**) The expression of FBXO9 proteins was identified by WB in OV cell lines. GAPDH was included as a reference gene. (**G**,**H**) Boxplot of the CERES project score for CRISPR KO of *UBD*, *FBXO9*, *BRCA1*, and *BRCA2* in 317 cancer cell lines (**G**) or 29 ovarian cancer cell lines (**H**). (**I**) The correlation between *FBXO9* CERES score and Ki-67 protein abundance in 8 ovarian cancer cell lines. Shown is the Spearman’s rank correlation coefficient.

The expression of *FBXO9* and *UBD* in our OV and control cohort described above was also assessed (Figure 1). The mRNA levels of *FBXO9* and *UBD* were determined using RT-qPCR. As expected, *FBXO9* was significantly down-regulated in OV (*n* = 30) compared to normal tissues (*n* = 20). However, *UBD* showed a mild but significant up-regulation (Figure 9A). This result, together with the correlation analysis with DDR pathways (Figure 7), suggested a key role for *FBXO9*, but *UBD*, during tumorigenesis of OV. In this sense, we further investigated the distribution of FBXO9 in ovarian tissues from our OV and control cohort, using IHC staining (Figure 9B). It was found that FBXO9 was localized to both cytoplasm and nucleus. OV lesions exhibited a significantly lower expression of FBXO9 as compared to normal ovary tissues (Figure 9C). Consistently, lower IRS score was obtained from OV primary lesion (4.368 ± 0.4773, *n* = 88) than normal tissue (7.159 ± 0.7809, *n* = 23, 5 samples were removed due to incorrect morphology). Additionally, the proportion of low FBXO9 sample (IRS > 5) was significantly higher for primary OV lesions (68%, 60/88) as compared with the normal ovary tissues (39.1%, 9/23) (Figure 9D). No statistically significant difference was found for clinicopathological characteristics between high FBXO9 and low FBXO9 expression OV patients (Table S3). In addition, consistent results were found in cell lines. Four OV cell lines (OVCAR-3, HEY, IGROV1, and HOC7) and one normal ovarian epithelial cell line IOSE80 were subjected to qPCR assay and three of the OV cell lines (OVCAR-3, HEY, and HOC7) demonstrated *FBXO9* mRNA expression significantly lower than normal cell line (Figure 9E). For protein level, the abundance of FBXO9 protein in 5 OV cell lines (HEY, SKOV-3, A2780, OVCAR-8, and TOV-21G) and the IOSE80 normal ovarian cell line were determined using WB assay. As a result, SKOV-3, OVCAR-8, and TOV-21G demonstrated decreased FBXO9 protein expression as compared with IOSE80 (Figure 9F). In addition, we analyzed the levels of K48-linked buiquitination in these five OV cell lines and found that it is not associated with FBXO9 expression (Figure S3B). This observation suggests the involvement of additional ubiquitination regulators besides FBXO9, highlighting the complexity of the ubiquitination process in OV.

To further explore the role of *FBXO9* in OV, the CRISPR knockout (KO) data on 317 cancer cell lines including 29 ovarian cancer cell lines from the CCLE database were analyzed. Interestingly, these data demonstrated that CRISPR KO of *FBXO9* led to a CERES project score of ~−0.2; this trend is similar to *BRCA1* and *BRCA2*, which are well-known tumor suppressors associated with DNA damage repair. By contrast, *UBD*, the other gene in our signature, exhibited a CERES score close to zero, indicating almost no dependency on cancer cells, ruling it out as a tumor suppressor (Figure 9G,H). Moreover, high Ki-67 expression is often correlated with malignant tumors, as it indicates rapid tumor cell proliferation. This may be attributed to the inactivation or functional impairment of tumor suppressor genes. Notably, our analysis indicated a negative correlation between *FBXO9* CERES score and Ki-67 protein abundance in 8 ovarian cancer cell lines (Figure 9I). In all, these findings suggested that *FBXO9* has the potential to function as a suppressor for the occurrence of OV.

## 4. Discussion

The high mortality of OV largely results from tremendous difficulties in diagnosis and prognosis prediction. OV is often discovered at advanced stages, which results in extremely low survival rates for patients [32]. Mounting evidence [33], including our observations (Figure 1), suggests that ubiquitination and deubiquitination play a vital role in ovarian oncogenesis. In the present study, we investigated the characteristics of UbRGs and identified a promising gene expression signature (specifically, *FBXO9* and *UBD*) based on UbRGs, which exhibited a close relationship with TME and predicted the response to immune-, chemo-, and targeted therapy for patients with OV. Moreover, our results implied that *FBXO9* might be a potential cancer suppressor for OV as it is significantly downregulated in primary OV lesions, and such tumor suppression is likely through interplay with DNA damage repair genes.

### 4.1. Advances of the UbRGs Signature for Prognostic Prediction in OV

The rationale of utilizing UbRGs for the prognostic prediction for OV is clear. Significant reduction in K48-linked ubiquitination was observed in primary OV lesions by IHC assays (Figure 1), suggesting that loss of ubiquitination activity might be associated with OV risk. Indeed, the association of ubiquitination with cancer has been studied for a long time. For example, proteasomal inhibitors that block the ubiquitin-proteasome pathway have been approved and used effectively in clinical trials for the treatment of multiple myeloma [34]. Previous studies have also established ubiquitin-related signatures for other cancer types such as lung adenocarcinoma, hepatocellular carcinoma, and glioma [35,36,37,38]. Hence, we developed a novel gene expression signature based on a screen on a collection of UbRGs and determined its prognostic significance in OV (Figure 2 and Figure 3). As this UbRG collection contains regulators of both ubiquitin and ubiquitin-like conjugations, the resulting signature consists of both ubiquitin E3 ligase and an ubiquitin-like modifier, indicating the complexity of the functions of ubiquitination and ubiquitination-like processes in OV.

Our gene signature consists of as few as two genes. A minimized number of involving genes would enhance the feasibility of developing practical diagnostic or prognostic tools and increase the likelihood of capturing the fundamental biological processes or pathways related to the studied phenomenon. Remarkably, this signature with only two genes demonstrated good predictive power as revealed by the ROC curve (Figure 4). Its predictive capability, estimated by the AUC value of ROC curve, is comparable with that of most OV prognostic signatures consisting of more genes, i.e., an aging-related gene signature with 8 genes [39] and is even better than a signature of ferroptosis-related long noncoding RNAs (lncRNAs) with 18 lncRNAs [40]. In addition, univariate and multivariate Cox regression analyses identified both age and risk score as independent prognostic factors for OV patients. When integrated with clinical characteristics, the model exhibited enhanced predictive efficacy compared to the standalone signature (Figure 5). Notably, among clinical characteristics integrated prognostic signatures, this signature exhibits a high AUC value of ROC curve, surpassing the predictive performance of both the endoplasmic reticulum stress-related gene signature and the m6A-related lncRNA signature in OV [41,42]. In summary, the ubiquitin-related signature showed a satisfactory prognostic value for patients with OV.

### 4.2. Potential Mechanisms Underlying Ubiquitination-Mediated OV Risk

Since we found that deficiency of ubiquitination, as reported by our signature, enhanced the risk of OV (Figure 4), a key issue would be how ubiquitination activity affects the development of tumors. Nowadays, it has become increasingly evident that tumors and immune cells cross-talk in a dynamic tumor immune microenvironment [43]. According to Figure 6, patients belonging to the low-risk group demonstrated elevated immune, stromal, and estimated scores, implying a potential lower tumor purity and thereby higher applicability of immunotherapy than individuals in the high-risk group [44]. There were notable differences in the immune cell composition between the high-risk and low-risk TME of OV. For instance, M1 macrophages, rather than M0 or M2, were more abundant in the low-risk group as compared to the high-risk group. This finding was in line with the notion that the transformation of M0 macrophages into M1 type significantly correlated with better prognosis, whereas M2 differentiation coincidences with worse outcomes [45], suggesting the involvement of TME macrophage differentiation in OV progression driven by ubiquitination. Moreover, we also noticed a decrease in the proportion of B cells in the low-risk group, consistent with previous studies that B cells have a positive or neutral effect on cancer progression [46]. Collectively, an increase in immune activity within TME was found in low-risk patients as compared to high-risk patients. This finding suggested that normal ubiquitination likely sustains the TME immune activity, thereby restraining tumor growth. In the meantime, our results indicated that the low-risk OV patients exhibited increased immune checkpoint expression, suggesting that these patients were likely more inclined to benefit from immunotherapies targeting immune checkpoints. Immunotherapy is currently not an effective treatment for ovarian cancer, but our results suggested that low-risk patients may have the opportunity to respond to certain immunotherapy such as immune checkpoint inhibitors targeting *CTLA4*.

Besides TME, the response to medical treatment significantly affects tumor survival. Despite the initial response to platinum-based chemotherapy and PARP inhibitors, a considerable portion of OV patients eventually experience relapse and drug resistance [47,48]. In the present study, the low-risk group showed enhanced responses to multiple commonly used anticancer medications (Figure 7). In other words, *FBXO9* and *UBD* likely enhance drug sensitivity in OV. Interestingly, in line with this finding, it has been reported that increased *UBD* levels in rectal cancer correspond to favorable treatment outcomes [49]. On the contrary, FBXO9 has been implicated in driving drug resistance in hepatocellular carcinoma [50]. This inconsistency is likely due to different cancer types, as FBXO9 was found to overexpress in hepatocellular carcinoma [50]. Therefore, it is reasonable to deduce that the activity of ubiquitination, marked by **FBXO9** and UBD expression, enhances the responsiveness to a range of drugs. The exact mechanism underlying the relationship between our risk model and the drug sensitivity is still unclear. However, ubiquitination might affect the protein profile in cancer cells, hence mediating either intrinsic signal pathways or the tumor microenvironment to induce drug resistance or sensitivity. Moreover, our risk model may influence current treatment regimens and pave the way for personalized therapies. Low-risk patients based on our model might be eligible for current commonly used drugs, but high-risk patients may require novel therapy or combined drugs. The prognostic signature has substantial potential in guiding clinical therapeutic decisions for OV.

### 4.3. Potential Functions of FBXO9 in OV

We found that *FBXO9*, together with *UBD*, constructs a promising signature for the prediction of prognosis for OV, but only *FBXO9* is significantly downregulated in OV, suggesting a strong involvement of *FBXO9* in OV. The *FBXO9* gene encodes the F-box only for protein 9, which is an E3 ubiquitin ligase belonging to the F-box family [51]. F-box proteins are critical components of the ubiquitin-proteasome system. FBXO9 protein is known to interact with a number of substrates and mediate their ubiquitination via E3 ubiquitin ligase activity, thereby influencing their stability and degradation [27,52]. Previous studies have shown that FBXO9 protein could function as either a cancer suppressor or an oncogene, depending on different cancer types [51]. For OV, the function of FBXO9 is still unclear. One previous study reported loss of heterozygosity (LOH) of the *FBXO9* gene in OV, which is associated with advanced tumor type, histological severity, tumor stage progression, and increased tumor metastasis [53]. However, whether LOH of *FBXO9* lead to loss of function of FBXO9 protein is questionable.

According to our observation, only *FBXO9*, but not *UBD*, exhibits high correlation with the expression of the relevant DDR gene signatures. This finding suggests a potential role for *FBXO9* in DDR (Figure 8). We also noticed that *FBXO9*, again not *UBD*, is downregulated in primary lesions of ovarian tissues for OV patients (Figure 9). The diminished expression of the *FBXO9* gene likely results in a decreased DNA repair and heightened genomic instability, which potentially contributes to tumorigenesis. In this sense, *FBXO9* might mimic *BRCA1* and *BRCA2*, the well-established cancer suppressors for OV that maintain genomic stability via facilitating DNA damage repair and are dysfunctional in a large portion of OV patients. In line with this idea, CRISPR KO data from the CCLE database revealed a similar CERES project score for *FBXO9* as that of *BRCA1/2*, suggesting that OV cancer cells exhibit comparable dependency on *FBXO9* as on *BRCA1/2* (Figure 9). These findings raised the possibility for *FBXO9* as a putative cancer suppressor for OV, but it still needs to be further testified in future studies.

According to our findings, greater attention must be paid to ubiquitination in OV studies in the future. Firstly, regarding the critical function of *FBXO9* in OV as revealed by our results, further studies are required to illuminate the mechanisms underlying *FBXO9* mediated OV risk, i.e., to identify the key ubiquitination substrate of *FBXO9* in OV. A preliminary screening based on the correlation between the level of various proteins and the transcriptional expression level of *FBXO9* from 617 TCGA-OV patient samples was performed, and 27 potential candidates of *FBXO9* were identified (Figure S6A,B). Surprisingly, *TELO2*, which encodes Tel2, a well-identified substrate for *FBXO9* in multiple myeloma [54], demonstrated no significance in this analysis. This result suggested that FBXO9 might mediate specific substrate(s) in OV, which require further studies to establish. Secondly, given the significant relationship between DNA damage repair and the *FBXO9*, further assessment of the efficacy of *FBXO9* on mediating HRR, NHEJ, BER, etc., is required to reveal the signal pathways connecting *FBXO9* ubiquitination with DDR activity in OV.

Meanwhile, our research has certain limitations. Firstly, as the drug resistance data are calculated only based on gene expression profile, we can only conclude the statistical significance of our risk score, whereas its clinical significance is still an open question and requires further investigation in future studies. Secondly, potential biases exist in bioinformatics and sample selection. For instance, when examining endoplasmic reticulum stress-related genes in ovarian cancer across different datasets, variations may arise due to bias in samole selection. However, it is noteworthy that some overlap exists among these datasets [41,55]. To address this issue, additional experiments are required for validation. Lastly, evidence supporting our speculation regarding the potential role of *FBXO9* as a tumor suppressor is still limited. To formally classify it as a tumor suppressor, further experimental validation using the KO animal model is indispensable. 

## 5. Conclusions

In conclusion, we found a reduction in K48-linked ubiquitination in OV lesions as compared to normal ovarian tissues. Moreover, we have identified and validated a signature related to ubiquitin, including *FBXO9* and *UBD*, that can assess prognosis, predict therapy response, and guide clinical treatment in OV. Through a comprehensive analysis, we discovered significant relationships between ubiquitin patterns and immune cell infiltration, indicating the potential for personalized treatment decision-making. Specifically, *FBXO9* might be a putative suppressor of ovarian cancer, at least partially via mediating DNA damage repair. These findings highlighted the involvement of ubiquitination in the occurrence and development of ovarian cancer.

## Data Availability

The datasets analyzed during the current study are available in the GEO database (https://www.ncbi.nlm.nih.gov/geo/, accessed on 11 September 2023), the UCSC Xena database (https://xenabrowser.net/, accessed on 11 September 2023), the GTEx database (https://www.gtexportal.org/, accessed on 11 September 2023), the STRING database (https://string-db.org/, accessed on 11 September 2023), and the GDSC database (https://www.cancerrxgene.org/, accessed on 11 September 2023), which is an updating, openly available resource for free download. The data of our cohort and the cell experiments that support the findings of this study are available on request.

## Supplementary Materials

The following supporting information can be downloaded at: https://www.mdpi.com/article/10.3390/biom13121724/s1, Figure S1: HE staining for detecting the origins of benign gynecological conditions in the ovaries. Figure S2: A flowchart of the study design for signature construction. Figure S3: K48-linked ubiquitination in OV patient tissues and cell lines. Figure S4: Mutation profile of the genome from low-risk versus high-risk OV patients. Figure S5: title: FBXO9 was involved in cell cycle, choline metabolism, and ECM-receptor interaction. Figure S6: 27 potential candidates of potential FBXO9 substrate were identified; Figure S7: Full size western blots used in this study. Table S1: Primer sequences for genes of interest. Table S2: The clinicopathological characteristics of low K48-linkage polyubiquitin patients versus high K48 patients. Table S3: The correlation between FBXO9 expression and clinicopathological characteristics of 88 OV patients.

## Abbreviations

OV	Ovarian cancer
DE-UbRGs	Differentially expressed ubiquitin-related genes
DUB	Deubiquitinating enzymes
GTEx	Genotype-Tissue Expression Project
TCGA	The Cancer Genome Atlas
GEO	Gene Expression Omnibus
TME	Tumor microenvironment
IRS	Immunoreactivity scores
TPM	Transcripts Per Million
GSEA	Gene set enrichment analysis
GDSC	Genomics of Drug Sensitivity in Cancer
GO	Gene ontology
KEGG	Kyoto Encyclopedia of Genes and Genomes
ROC	Receiver operating characteristic
UbRGs	Ubiquitin-related genes
PPI	Protein–protein interaction
OS	Overall survival
mRNA	Message RNA
IHC	Immunohistochemistry
TMB	Tumor mutation burden
AUC	Area under the curve
IC50	Half-maximal inhibitory concentration
DDR	DNA damage repair
BER	Base excision repair
HRR	Homologous Recombination repair
MMR	Mismatch repair
NHEJ	Non-homologous end joining
LncRNAs	long noncoding RNAs
LASSO	least absolute shrinkage and selection operator
K-M	Kaplan–Meier
CRISPR	Clustered Regularly Interspaced Short Palindromic Repeats
CCLE	Cancer Cell Line Encyclopedia
KO	Knowout
HE	Hematoxylin and Eosin
BP	Biological Process
CC	Cellular Component
MF	Molecular Function
