# Supplementary material for: Identification of a Prognostic Signature for Ovarian Cancer Based on Ubiquitin-Related Genes Suggesting a Potential Role for FBXO9
Source: Biomolecules. 2023 Nov 30;13(12):1724. doi: 10.3390/biom13121724 (PMC10742228; doi:10.3390/biom13121724)
Supplement: Supplementary file 1 [file biomolecules-13-01724-s001.zip › supplement Table.pdf]

Table S1. Primer sequences for genes of interest

Genes	Primer sequence (5'-3')
FBXO9	5'- GATGGTGATGGCGTTGGAAACAG -3'
	5'- TGTGAGTTGCTGCTGGAAGTAGG -3'
GAPDH	5'- GAAGGTGAAGGTCGGAGTC -3'
	5'-GAAGATGGTGATGGGATTTC -3'
UBD	5'- GGTGCGAAGGTCCAGCTCAG -3'
	5'- TGCCATCATCTTCCCATCTTCCAG -3'

Table S2. The correlation between K48-linkage polyubiquitin chain abundance and clinicopathological characteristics of 83 OV patients

	low K48 (IRS<5)	high K48 (IRS<5)	P Value
Age (n,%)			0.6607
≤55	17 (42.5%)	21 (48.84%)	-
>55	23 (57.5%)	22 (51.16%)	-
FIGO stage (n,%)			0.6530
I-II	15 (37.5%)	14 (32.56%)	-
III-IV	25 (62.5%)	29 (67.44%)	-
Pathology stage (n,%)			0.6627
I-II	21 (52.5%)	20 (46.51%)	-
III	19 (47.5%)	23 (53.49%)	-
Histology type (n,%)			0.6219
Serous	27 (67.5%)	30 (75%)	-
Other types	13 (32.5%)	12 (25%)	-
Tumor diameter (n,%)			0.999
<10 cm	17 (56.67%)	24 (55.81%)	-
≥10 cm	13 (43.33%)	19 (44.19%)	-
Serum CA-125 (n,%)			0.055
<35 U/ml	17 (56.67%)	14 (32.56%)	-
≥35 U/ml	13 (43.33%)	29 (67.44%)	-

Abbreviation: FIGO stage Federation of International of Gynecologists and Obstetricians stage

Table S3. The correlation between FBXO9 expression and clinicopathological characteristics of 88 OV patients

	low FBXO9 (IRS<5)	high FBXO9 (IRS<5)	P Value
Age(n,%)			0.119
≤55	26 (44.83%)	19 (63.33%)	-
>55	32 (55.17%)	11 (36.67%)	-
FIGO stage(n,%)			0.999
I-II	26 (44.83%)	14 (46.67%)	-
III-IV	32 (55.17%)	16 (53.33%)	-
Pathology stage(n,%)			0.999
I-II	34 (58.62%)	17 (33.33%)	-
III	24 (41.38%)	13 (35.14%)	-
Histology type(n,%)			0.6518
Serous	36 (62.07%)	17 (56.67%)	-
Other types	22 (37.93%)	13 (43.33%)	-
Tumor diameter(n,%)			0.3628
<10 cm	36 (62.07%)	15 (50%)	-
≥10 cm	22 (37.93%)	15 (50%)	-
Serum CA-125(n,%)			0.6540
<35 U/ml	27 (46.55%)	16 (53.33%)	-
≥35 U/ml	31 (53.45%)	14 (46.67%)	-

Abbreviation: FIGO stage Federation of International of Gynecologists and Obstetricians stage
