# Supplementary figures and images for: Identification of a Prognostic Signature for Ovarian Cancer Based on Ubiquitin-Related Genes Suggesting a Potential Role for FBXO9
Source: Biomolecules. 2023 Nov 30;13(12):1724. doi: 10.3390/biom13121724 (PMC10742228; doi:10.3390/biom13121724)

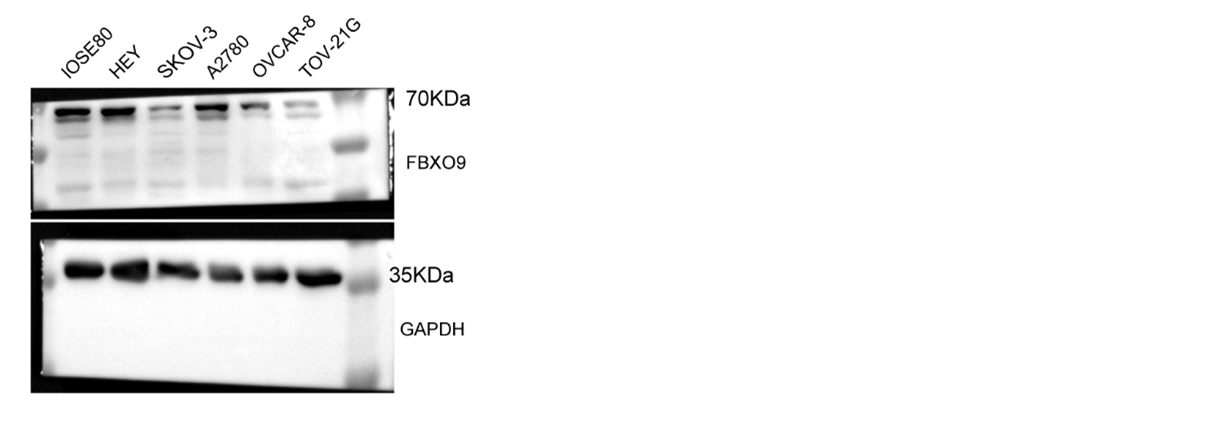

Supplement: Supplementary file 1 [file biomolecules-13-01724-s001.zip › biomolecules-2633997-original images.tif]

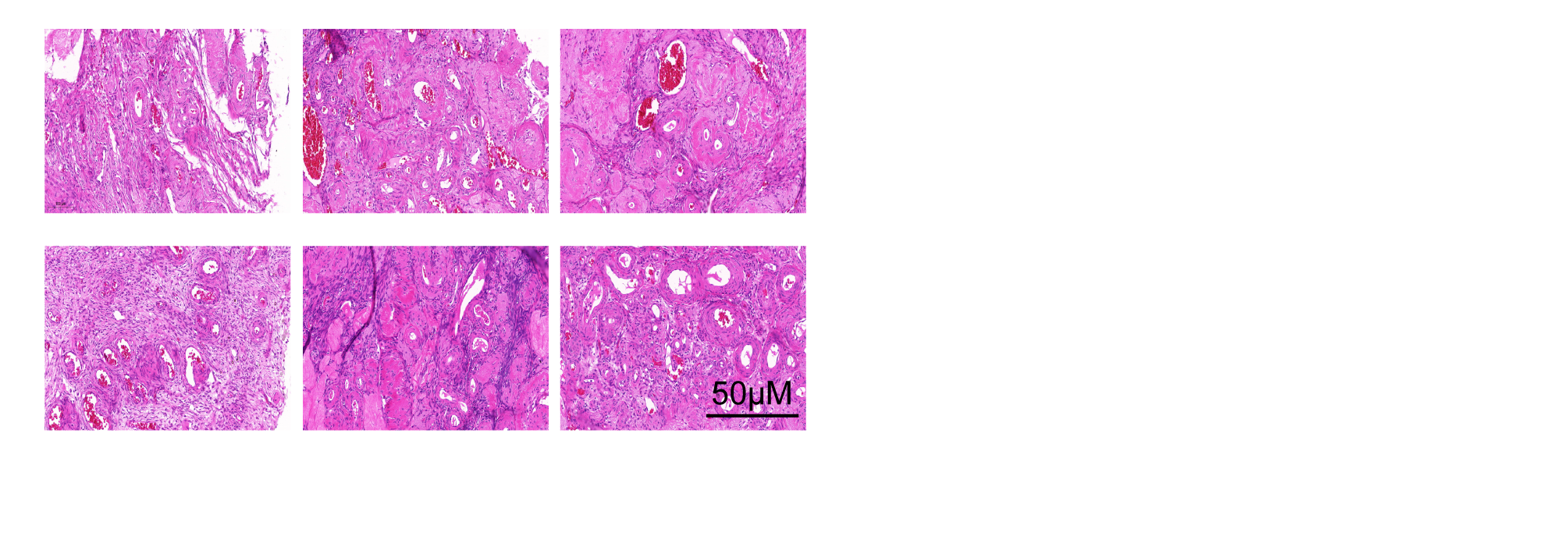

Supplement: Supplementary file 1 [file biomolecules-13-01724-s001.zip › supplement figure 1.tif]

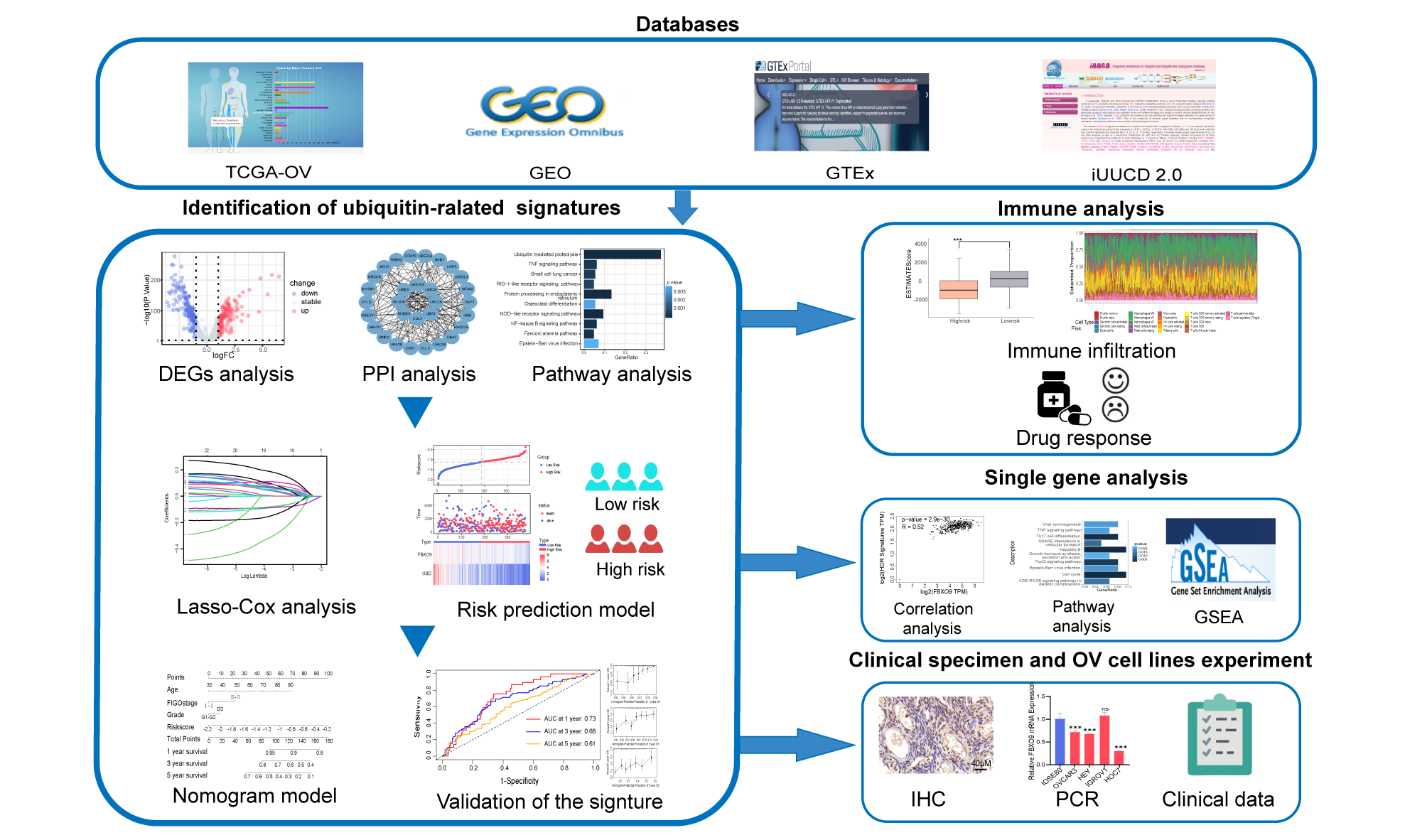

Supplement: Supplementary file 1 [file biomolecules-13-01724-s001.zip › supplement figure 2.tif]

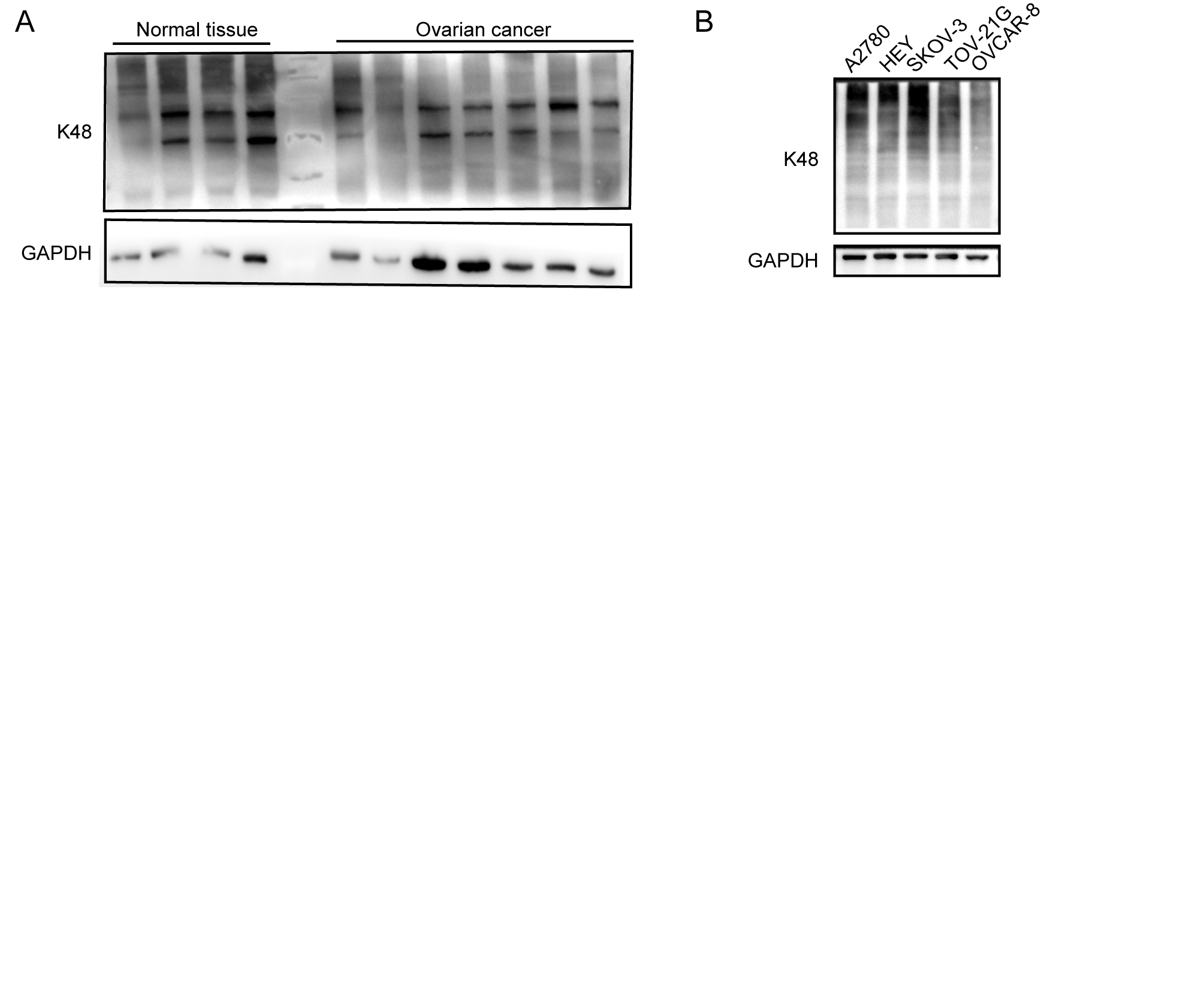

Supplement: Supplementary file 1 [file biomolecules-13-01724-s001.zip › supplement figure 3.tif]

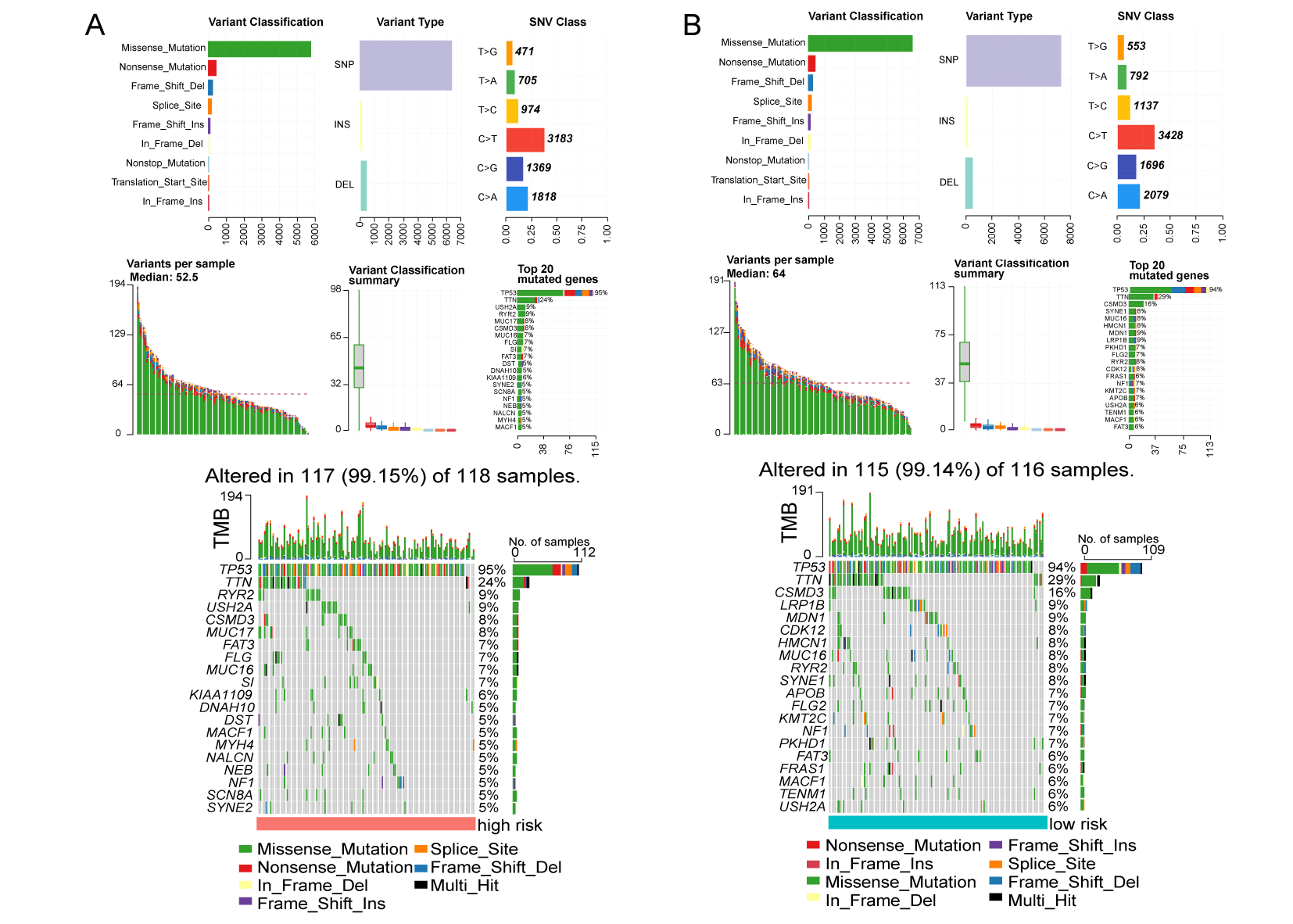

Supplement: Supplementary file 1 [file biomolecules-13-01724-s001.zip › supplement figure 4.tif]

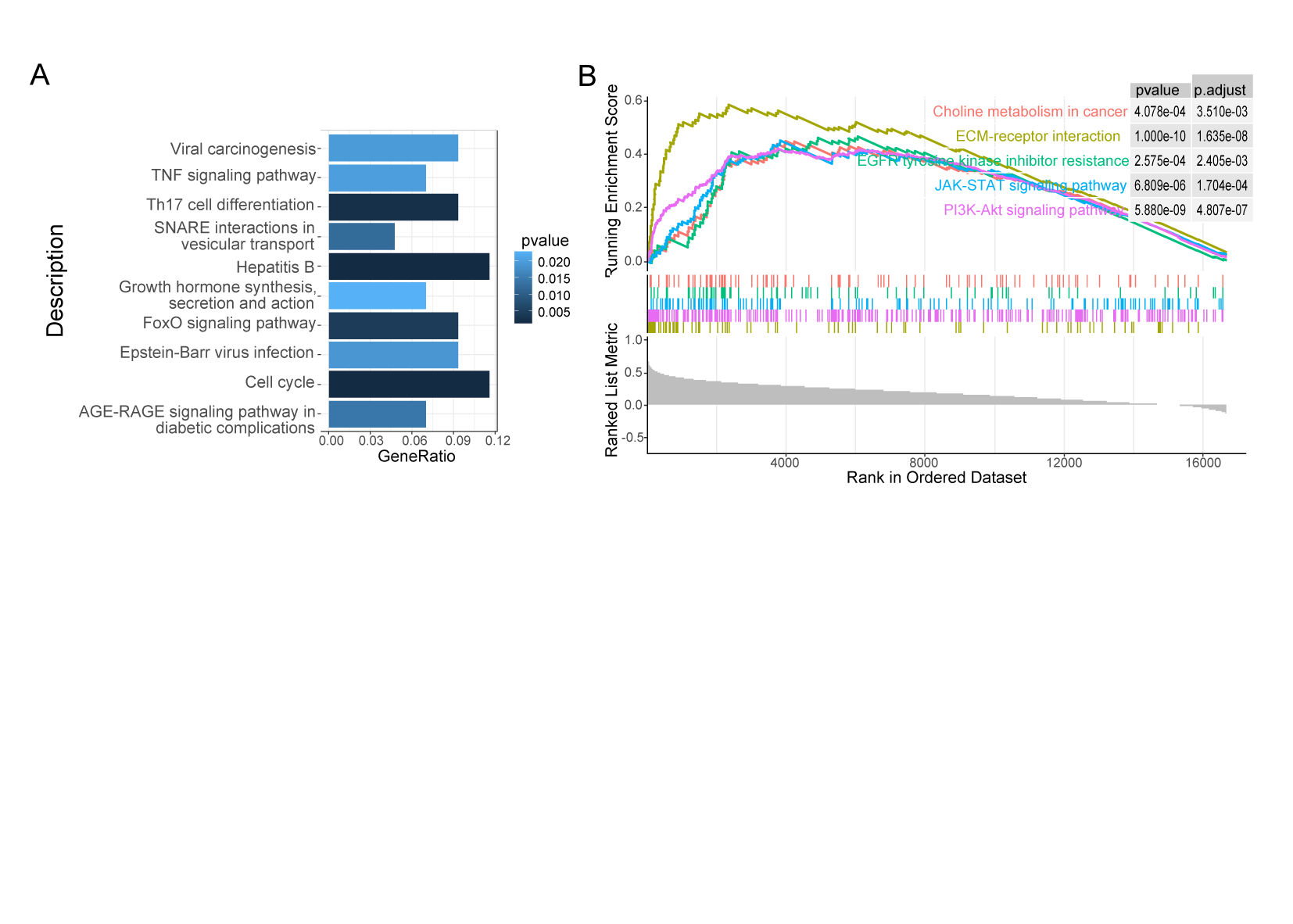

Supplement: Supplementary file 1 [file biomolecules-13-01724-s001.zip › supplement figure 5.tif]

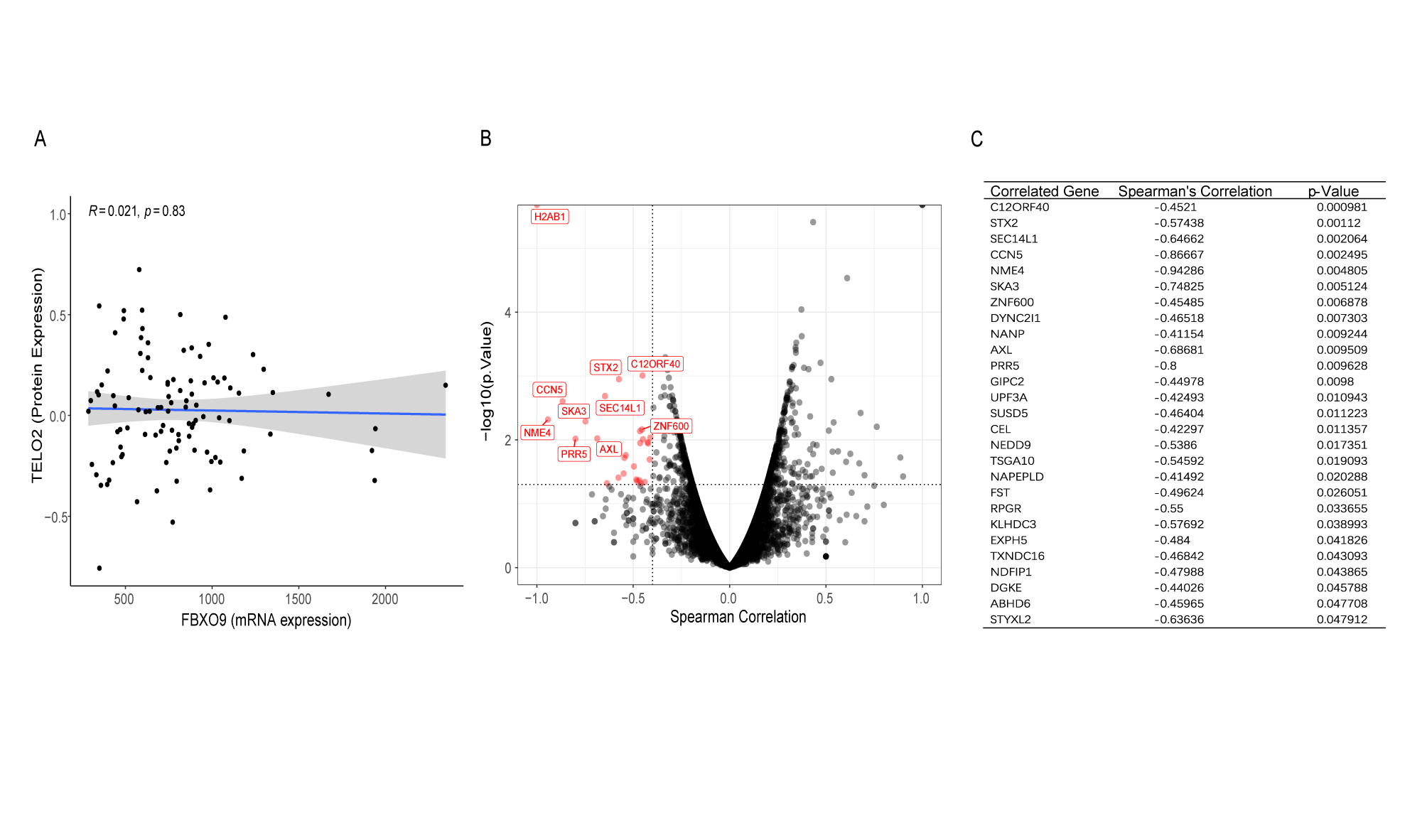

Supplement: Supplementary file 1 [file biomolecules-13-01724-s001.zip › supplement figure 6.tif]

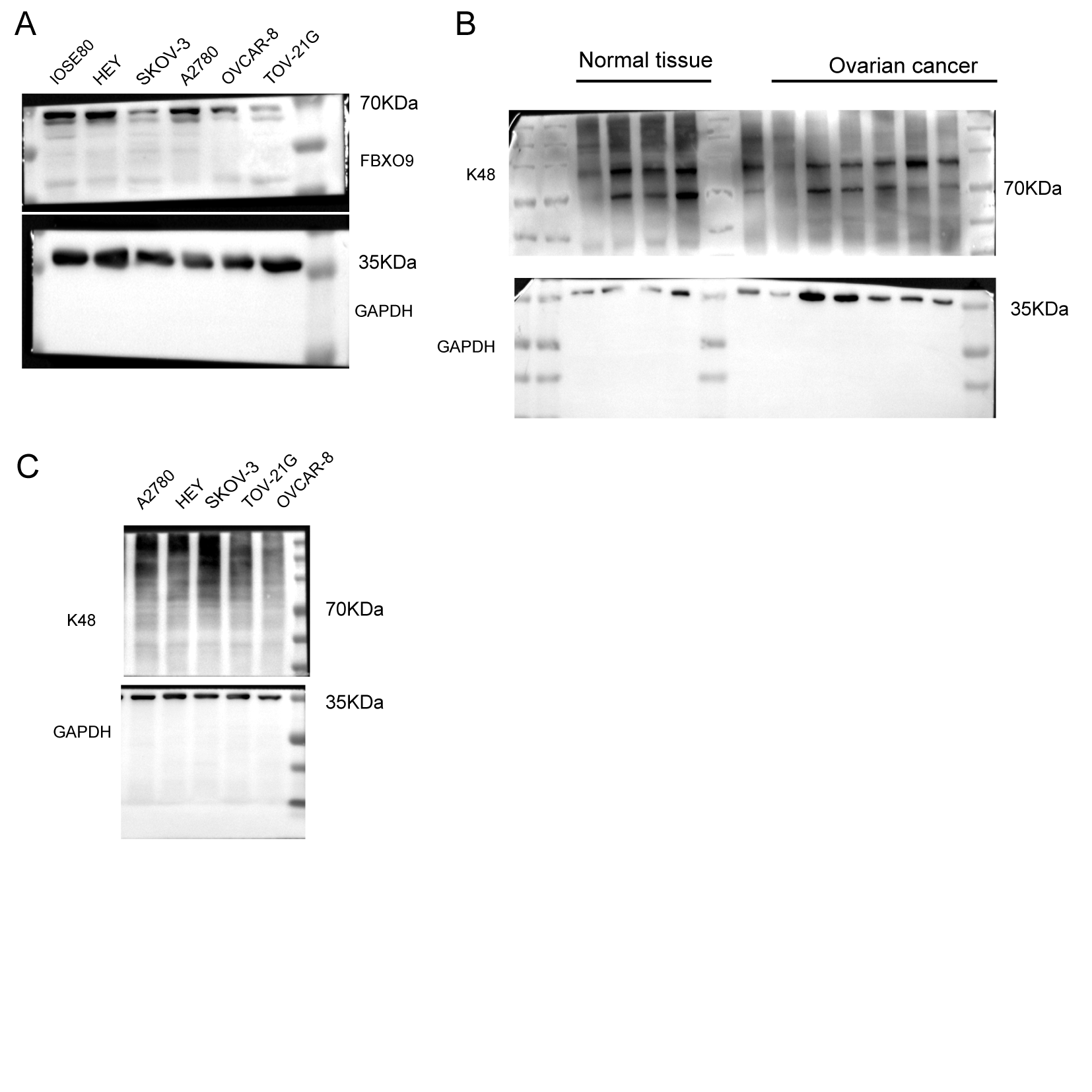

Supplement: Supplementary file 1 [file biomolecules-13-01724-s001.zip › supplement figure 7.tif]
